# Whole Heart Coronary Imaging with Flexible Acquisition Window and Trigger Delay

**DOI:** 10.1371/journal.pone.0112020

**Published:** 2015-02-26

**Authors:** Keigo Kawaji, Murilo Foppa, Sébastien Roujol, Mehmet Akçakaya, Reza Nezafat

**Affiliations:** Department of Medicine, Beth Israel Deaconess Medical Center and Harvard Medical School, Boston, Massachusetts, United States of America; University of Louisville, UNITED STATES

## Abstract

Coronary magnetic resonance imaging (MRI) requires a correctly timed trigger delay derived from a scout cine scan to synchronize k-space acquisition with the quiescent period of the cardiac cycle. However, heart rate changes between breath-held cine and free-breathing coronary imaging may result in inaccurate timing errors. Additionally, the determined trigger delay may not reflect the period of minimal motion for both left and right coronary arteries or different segments. In this work, we present a whole-heart coronary imaging approach that allows flexible selection of the trigger delay timings by performing k-space sampling over an enlarged acquisition window. Our approach addresses coronary motion in an interactive manner by allowing the operator to determine the temporal window with minimal cardiac motion for each artery region. An electrocardiogram-gated, k-space segmented 3D radial stack-of-stars sequence that employs a custom rotation angle is developed. An interactive reconstruction and visualization platform is then employed to determine the subset of the enlarged acquisition window for minimal coronary motion. Coronary MRI was acquired on eight healthy subjects (5 male, mean age = 37 ± 18 years), where an enlarged acquisition window of 166–220 ms was set 50 ms prior to the scout-derived trigger delay. Coronary visualization and sharpness scores were compared between the standard 120 ms window set at the trigger delay, and those reconstructed using a manually adjusted window. The proposed method using manual adjustment was able to recover delineation of five mid and distal right coronary artery regions that were otherwise not visible from the standard window, and the sharpness scores improved in all coronary regions using the proposed method. This paper demonstrates the feasibility of a whole-heart coronary imaging approach that allows interactive selection of any subset of the enlarged acquisition window for a tailored reconstruction for each branch region.

## Introduction

High-resolution MRI of the heart for applications such as coronary artery imaging provides unique challenges among most anatomical imaging due to both cardiac and respiratory motion. Recent advances in hardware and software MR technologies have allowed whole-heart imaging, specifically for the visualization of the coronary arteries, to be performed in a clinically feasible scan time at both 1.5T and 3T [1–7]. In practice, high-resolution whole-heart and targeted cardiac imaging utilize an ECG-gated k-space segmented acquisition strategy, which samples the 3D k-space volume only during the quiescent period of the cardiac cycle. For all of these methods, a correctly timed trigger delay (TD) to synchronize the segmented k-space acquisition with the quiescent period of the cardiac cycle is essential. Therefore, a prior scout cine acquisition is typically employed prior to the coronary imaging sequence to determine the optimal TD value for each individual [8]. However, subjects demonstrating heart-rate variability may present image quality degradation due to inaccurate TD timing errors [9]. An accurate timing determination for whole-heart coronary imaging can be difficult in such subjects, as the heart rate can vary from beat to beat over the duration of a long scan, or change significantly in the presence or absence of breath-holding. In addition to the per-patient and physiological variability, the optimal period quiescent period with minimal motion for each artery branch may vary by anatomical position [10,11]. Therefore, a single acquisition window may not necessarily be optimally timed for each coronary artery branch and region.

To overcome these challenges, multiphase acquisition strategies with non-Cartesian sampling schemes have been incorporated into coronary imaging. Stehning *et al*. have proposed a segmented 3D radial stack-of-stars acquisition approach that generated four consecutive volumes at each temporal sub-window [12], combining these sub-images to improve image quality. Additionally, several other studies have explored coronary acquisition over multiple temporal phases using k-space segmented strategies [6,13] or high-resolution 3D cine approaches [14–17] with radial or spiral sampling patterns. In all of the aforementioned multiphase 3D volumetric imaging approaches, the temporal window resolution is defined as the duration of each equally spaced temporal phase, and is not inherently designed for retrospective processing of any desired temporal subset within the acquisition window.

In this work, we propose an alternative acquisition scheme whose reconstruction window can be retrospectively selected by the operator in an interactive manner as any subset of the acquisition window (with a minimum window size of ~30 ms) and temporal resolution of a single readout (~4 ms). A radial stack-of-stars sampling based on golden angle (GA) radial interleaving is processed retrospectively to determine the period of minimal coronary artery motion within the acquired temporal window, which additionally allows tailoring of the optimal reconstruction window to individual branches and regions of each coronary artery.

## Materials and Methods

### 3D Stack-of-Stars K-space Segmented Sequence and its Optimal K-space Distribution

**Fig 1:**
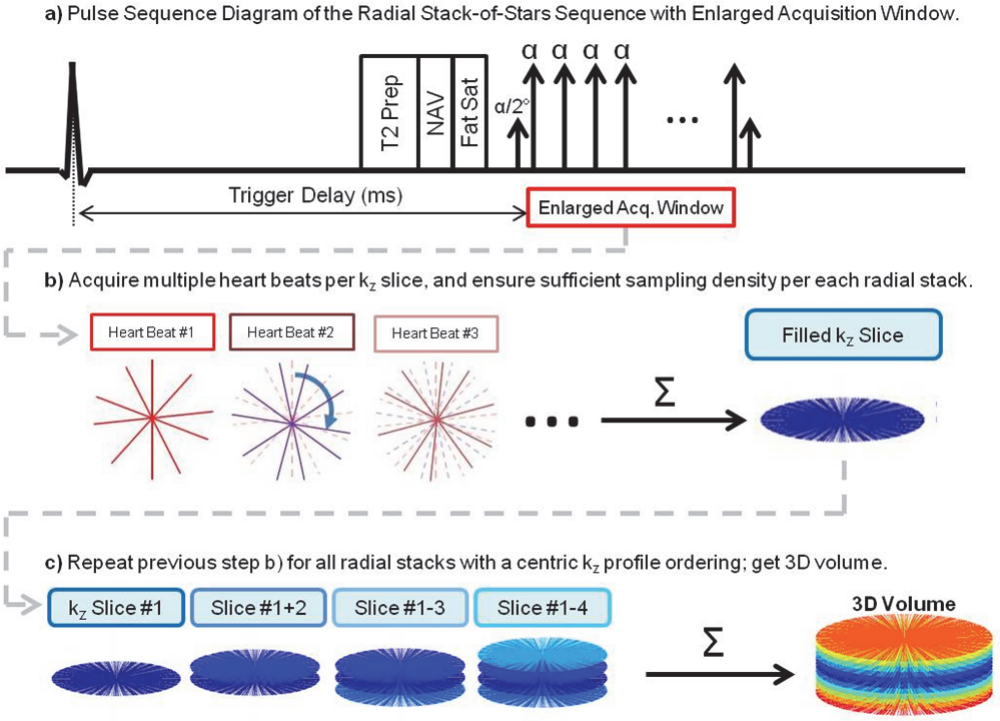
This diagram demonstrates the sampling scheme with the enlarged acquisition window: a) is the pulse sequence diagram that acquires an enlarged acquisition window. b) shows each k_z_ plane being repeatedly acquired over a fixed number of heart beats. c) shows a centric k_z_ ordering of the radial stacks to fill the 3D k-space volume.

**Fig. 1** shows the schematics of the proposed sampling scheme. Our proposed approach uses an ECG-gated, k-space segmented 3D balances steady-state free precession (b-SSFP) enlarged acquisition window that utilizes a radial stack-of-stars sampling strategy with a customized rotation angle *θ*. We acquire each k_z_-plane one at a time in a centric order, instead of all k_z_ slices for a particular radial spoke position at once prior to applying a rotation angle.

Typically, for radial imaging strategies that yield a single volume, radial spokes are acquired in such way that that the resulting k_z_ plane is uniformly distributed; thus, if n radial spokes are acquired on each plane, then a rotation angle that ensures uniform distribution satisfies the equation *θ* = j ∙ 180° / n, where j is an integer such that the greatest common divisor of j and n is 1. *θ* is often set to 180° / n, (i.e. j = 1), where a sequential linear sweep is achieved through the plane.

An alternate scheme uses a golden angle (GA) rotation angle where *θ* is set to 111.246°. This scheme allows for retrospective reconstruction of the data with different temporal resolution, and has been utilized in dynamical imaging approaches with both radial and spiral trajectories in 2D for cardiac real-time imaging [18,19]; in 3D for the liver [20–22], brain [23], and the heart [24–26]; as well as in both 2D and 3D for imaging the vocal chords [27–31].

**Fig 2:**
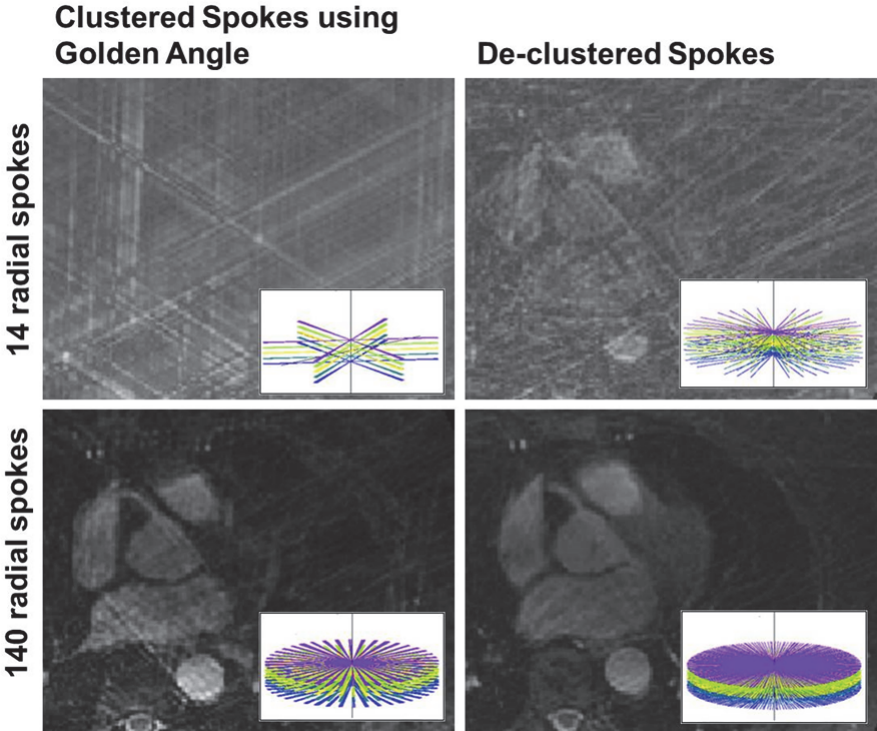
A counter-example showing that using a Golden Angle radial spoke rotation (left column) may not be optimal in a 3D stack-of-stars sequence. The top row shows the k-space spokes and its gridding reconstruction performed from data from a single echo readout acquired over 14 consecutive heartbeats, while the bottom row shows the spoke distribution from 10 consecutive echoes. Using a different angle (right column; 15.89°), a more uniform distribution of the spokes is achieved. The spoke distribution is sensitive to the rotation angle, the number of TFEs per each heart beat, and the number of heart beats required to fill each k_z_ stack. Written informed consents were obtained from the subjects for publication of all image data.

In this study, we seek to incorporate an acquisition strategy that enables a flexible reconstruction from any subset of the acquisition window. In a 3D stack-of-stars sequence acquired in an ECG-gated and k-space segmented scheme, a constant *θ* rotation is applied between each spoke, and this holds true between the final radial spoke of the acquisition window and the first spoke in the subsequent heartbeat. Therefore, the acquired k-space distribution becomes sensitive to the number of projection lines, or the Turbo Field Echoes (nTFE) in each heart beat as the acquired radial spokes over multiple heartbeats are temporally indexed modulo of nTFE. Furthermore, to achieve flexible retrospective selection and reconstruction, any subset of this temporal window must have the sorted k-space spokes to be distributed as uniformly as possible for minimal artifacts; and this is not necessarily achieved by using *θ* = GA, as shown in **Fig. 2**. In this figure, the GA interleaving results in clustering of the spokes within a small temporal window when the acquisition window is set to 48 TFEs (left column). We also show that an improved distribution is feasible by using a different rotation angle (right column), and this hold true whether we use a reconstruction window of a single TR, or with 10 consecutive TRs, both containing spokes acquired over multiple repeated heartbeats (n = 14). In the subsequent sections, we will show how this is achieved.

The optimal k-space distribution over any temporal window subset can be examined as a function of 4 parameters; the rotation angle *θ* between consecutive radial spokes, the number of projection lines per heart beat (nTFE), the number of heartbeats per k_z_ slice (nHB), and the number of projections in each heartbeat used for reconstruction, i.e. the reconstruction window (nRCW).

An empirical simulation-based approach was developed to assess and optimize the radial coverage over a flexible subset of the temporal window. In this study, the repeated number of heartbeats was fixed to nHB = 14, as this value corresponded to a 100% Nyquist sampling density over an 80 ms temporal window. In this optimization, we seek an optimization to enforce both even spacing of the radial spokes over a flexible range of the reconstruction window (nRCW), and to minimize the clustering of spokes in any sector of the reconstruction window. Accordingly, we define the objective function to minimize the following:

$$C(\text{nTFE, nHB})=\underset{\theta }{\text{arg}\;\text{min}}\sum_{\text{nRCW=Nmin}}^{\text{Nmax}}∥\Delta \Theta_{\theta ,\;\text{nTFE}}^{\text{nHB},\;\text{nRCW}}-\Delta \Theta_{LI,\;\text{nTFE}}^{\text{nHB},\;\text{nRCW}}{∥}_{2}^{2}+\lambda \cdot \max(|\Theta_{\theta ,\;\text{nTFE}}^{\text{nHB},\;\text{nRCW}}-\Theta_{LI,\;\text{nTFE}}^{\text{nHB},\;\text{nRCW}}|)$$

where $\Theta_{\theta ,\;\text{nTFE}}^{\text{nHB},\;\text{nRCW}}$ is a vector corresponding to the angles of the nHB ∙ nRCW radial spokes acquired within the reconstruction window over multiple heartbeats when a *θ* rotation is used between subsequent spokes, $\Theta_{LI,\;\text{nTFE}}^{\text{nHB},\;\text{nRCW}}$ is the same vector of radial angles for the linear case with a 180°/(nHB ∙ nRCW) rotation between subsequent spokes, and ∆Θ is the angle gap vector, which is the first differential of the Θ vector. We note that in this formulation, the reconstruction window is a subset of the acquisition window, hence nRCW ≤ nTFE. Additionally, $\Theta_{\theta ,\;\text{nTFE}}^{\text{nHB},\;\text{nRCW}}$ is invariant to the reconstruction window position within the acquisition window, and its derivation is shown in the Appendix. For the final objective function determination, all range of nRCW values was accounted for by taking the summation of nRCW between Nmin and Nmax.

In this optimization, the first (left) term enforces uniform spacing, while the second (right) one minimizes clustering. We note the right term is not rotation invariant, thus the first element of angle of $\Theta_{\theta ,\;\text{nTFE}}^{\text{nHB},\;\text{ nRCW}}$ was chosen such that the $\Theta_{\theta ,\;\text{nTFE}}^{\text{nHB},\;\text{ nRCW}}$ was best aligned with $\Theta_{LI,\;\text{nTFE}}^{\text{nHB},\;\text{nRCW}}$, by minimizing $||\Theta_{\theta ,\;\text{nTFE}}^{\text{nHB},\;\text{nRCW}}-\Theta_{LI,\;\text{nTFE}}^{\text{nHB},\;\text{nRCW}}|{|}_{2}^{2}$.

In this simulation, for each candidate *θ* at a fixed (nTFE, nHB) pair, the reconstruction window size nRCW was varied between Nmin = 7 (98 spokes; 44% sampling density) and Nmax = 25 (350 spokes; 156% sampling density), which corresponded to a temporal window of duration 32–115 ms at TR = 4.6 ms. The λ value was empirically set to 0.7, which yielded the least clustering across all examined nTFEs.

In order to determine how well the GA/n_optimal_ distributed the radial spokes among all possible input angles, an exhaustive numerical optimization using equation [1] was also performed. For this optimization, all *θ* values between [0.01, 179.99] were examined for the even nTFEs between 32 and 64 and nHB = 14, and were sorted according to their corresponding cost values from the objective function. The *θ* = GA/n_optimal_ was expressed as a percentile rank among all possible angles.

### Real-Time Visualization of Platform Design

An in-house accelerated reconstruction platform was developed in MATLAB for the visualization, and in C++ and NVIDIA CUDA for real-time non-Cartesian radial k-space gridding reconstruction. The raw k-space data and scanner parameters are used to automatically prepare the data for visualization on the platform, which consists of the following four steps: 1) temporal indexing and k-space look-up table generation, 2) coil compression, and 3) 3D volume data loading onto the CPU memory.

In step 1, the raw k-space data is first indexed into the following coordinate system [k_x_, k_y_, k_z_, t], and a look-up table is generated for rapid referencing of k-space from any desired temporal window. In step 2, a coil compression method proposed by Zhang *et al*. was employed to load the 3D k-space volume from 32 channels onto our standard workstation running the visualization platform; data was compressed from 32 to 8 compressed channels [32]. This compression method, which was originally proposed for Cartesian sampling, was feasible in our approach as the k_z_-direction is fully sampled and thus was used as the spatially transformed direction to generate the hybrid k-space domain required for this approach. An additional Fourier transform in the k_z_ direction is applied during this step, yielding an equivalent set of k-space values and their coordinates in the [k_x_, k_y_, z, t] space, where z corresponds to the axial slice. In step 3, the processed data is finally mounted onto the CPU memory for visualization. Mounted data is stored as temporally indexed and channel-compressed k-space values, and coordinate position. The operator uses the GUI to select the z-slice and temporal window, and the developed GPU reconstruction engine performs the gridding operation of the selected non-Cartesian k-space data, and outputs the reconstructed image for immediate display onto the GUI panel. The gridding operation is parallelized and performed on a per-point basis, where gridded data from each GPU kernel is accumulated onto the final Cartesian grid using a dedicated GPU atomic addition operation [33]. Image reconstruction is performed as a sequence of four sub-steps: a) zero-padding, b) inverse FFT, c) Cropping to the correct FOV, and d) Roll-off correction. After completing these steps, the reconstructed image is mounted back to the CPU memory, and is returned to the MATLAB GUI.

For the GPU-gridding step, a fixed Kaiser-Bessel gridding kernel size of W = 5.5 and a default oversampling factor of 1.25 were chosen in this study, for which the corresponding gridding shape *beta* parameter set to 9.9981 per approximation method proposed by Beatty et al. [34].

The performance of each GPU reconstruction substep was evaluated using different input k-space parameters typically acquired in high-resolution coronary MRI, and the measured times were averaged over 100 operations. For comparison, the reconstruction was also performed and evaluated using the NUFFT package [35] provided in MATLAB, which requires an initial preparation step.

### In Vivo Imaging

All scans were performed on a 1.5T Philips Achieva MR system using a 32-channel cardiac array under an institutional review board (IRB) approved protocol (IRB No. 2001-000793; approved by the Committee on Clinical Investigation of Beth Israel Deaconess Medical Center, Boston, MA). Written informed consents were obtained from all subjects prior to imaging, allowing use of all acquired MR data in our studies. Eight healthy subjects (5 male, mean age = 37 ± 18 years) were scanned. The ECG-gated 3D radial stack-of-stars SSFP coronary MRI was performed approximately 2 minutes post-contrast injection (0.2 mmol/kg, Gadobenate Dimeglumine) with the following parameters: TR = 4.6 ms, TE = 1.8 ms, FA = 100°, FOV = 300 x 300 x 80–120 mm^3^. Spatial resolution = 1.3 x 1.3 x 2.0 mm^3^. The subject-specific gating delay was determined from a breath-held 2D cine, and was adjusted 50 ms prior to the determined quiescent period. The prolonged acquisition window size was set to 220 ms for nTFE = 48 for subjects with a heart rate (HR) < 65, while a smaller window of 166 ms (nTFE = 36) was used for HR that exceeded 65. Accordingly, a custom radial angle of *θ* = 15.89 (nTFE = 48) and *θ =* 9.27 (nTFE = 36) was used (from Table 1), respectively. The coronary MRI sequence used a T2Prep pulse with an echo time of 50 ms, a diaphragmatic navigator with a 7 mm gating window with slice-tracking (tracking factor = 0.6), fat saturation, and a single half-alpha ramp up pulse, which was selected to minimize the time between all magnetization preparation pulses with volumetric imaging over the prolonged acquisition window to maximize the fat saturation over the enlarged acquisition window. Physiological parameters were extracted from the cine and the coronary imaging sequences. The optimal temporal reconstruction window for the right coronary artery (RCA) and the left anterior descending artery (LAD) were retrospectively determined manually by an experienced cardiologist using the developed visualization tool on a clinical workstation. The interactive assessment for the selection of the optimal coronary vessel visualization allowed selection of: 1) z-slice in the image display for scrolling through the 3D volume, 2) zoom, 3) manipulation of the window level, and 4) adjustment of both the start and end of the temporal window. A typical workflow was as follows: the standard 120 ms window at the TD was first examined; the temporal window was then adjusted by the operator to yield improved visualization of the examined artery branch region. The window that yielded a notably better vessel region was then examined for its sharpness.

**Table 1:** Simulation Results indicating the optimal GA/n rotation angle in a k-space segmented radial stack of starts sequence that provides uniform distribution over a range of reconstruction windows.

nTFE	GA/n_optimal_ with λ = 0.7	Percentile (%) rank per 18000
32	55.62 (GA/2)	5
34	13.91 (GA/8)	10
36	9.27 (GA/12)	0.5
38	55.62 (GA/2)	12
40	7.42 (GA/15)	3
42	37.08 (GA/3)	3
44	18.54 (GA/6)	12
46	13.91 (GA/8)	10
48	15.89 (GA/7)	5
50	13.91 (GA/8)	3
52	10.11 (GA/5)	7
54	8.56 (GA/13)	0.01
56	10.11 (GA/11)	3
58	7.95 (GA/14)	5
60	13.91 (GA/8)	7
62	12.36 (GA/8)	1
64	9.27 (GA/2)	5

In Bold—nTFE (36 = 166 ms window, 48 = 220 ms window) and angles used in this study.

All image reconstruction at the desired temporal window was performed offline on the in-house visualization platform described previously. For assessment of the coronary vessel sharpness, 3D volumes were reconstructed to 0.65 x 0.65 x 2.0 mm^3^ resolution with zero-filled interpolation using the platform. Vessel sharpness was measured using the SoapBubble Tool [36], a semi-automated two-step approach that first performs software-assisted delineation of a multi-planar reformat of the visualized artery region, followed by an fully automated sharpness calculation on the reformatted plane using Deriche edge detection [37]. Sharpness measurements were measured by an independent operator using the SoapBubble tool. The measurements were repeated twice for intra-observer variability. This tool was also used to measure the luminal signal-to-noise ratio (SNR), surrounding myocardium SNR, and the lumen-to-myocardium contrast-to-noise ratio (CNR), where the lumen ROI was prescribed on the targeted artery region after the multi-planar reformat, and the myocardium ROI was prescribed on the myocardial tissue adjacent to the lumen ROI. Background air was used for noise measurement.

All statistics were performed using Microsoft Excel (Microsoft, Redmond, WA). The Student’s t-test was used for the comparison of all measurements.

## Results

### Simulation of K-space distribution optimization

**Fig 3:**
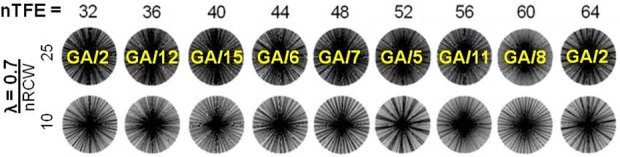
Best GA/n reconstructions for nTFE = [32, 36, … 64], when λ = 0.7, and n = [1,15]. The extent of even spoke distribution and absence of clustering varied between cases. For example, the results with nTFE = 52 remained notably clustered compared to the other nTFE cases even after the proposed optimization, as spoke banding can be seen at nRCW = 10.

**Fig. 3** shows the best GA/n reconstructions for nTFEs between 32 and 64 (in step sizes of 4) when λ was set to 0.7; which was manually determined to provide a de-clustered selection of the spokes in all tested cases for all even nTFEs between 32 and 64.

Compared to other GA/n cases, these cases yielded both evenly distributed and de-clustered results after the proposed optimization. The look-up table was generated for all examined nTFEs = [32, 34, 36, … 64], and is reported in Table 1, along with the percentile rank out of 18000. These cases were within the top 12 percentile. A look-up-table-derived GA/n angle reported in Table 1 provided a reasonable coverage of the k_x_-k_y_ planes with good image quality even under undersampled conditions, as the proposed objective function employed in this study accounts for both the optimal angle gap spacing and clustering of radial spokes within a sector of the k_x_-k_y_ plane. The reported rotation angles offer reasonable approximations for a range of nTFEs when nHB is fixed to 14, and images were acquired using the reported table values for nTFE = 36 and 48 in the subsequent sections.

### Visualization Platform Evaluation

**Fig 4:**
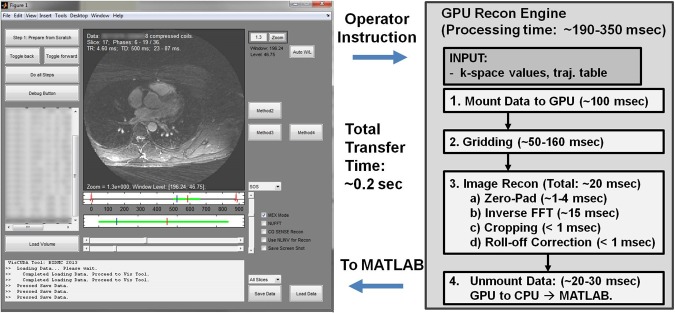
Screenshot of graphical user interface (left side) and workflow of GPU reconstruction (right side). Computation times are reported for each step, requiring a total of 190–350 milliseconds for the GPU operation to perform. An additional 0.2 ms is required for total latency to transfer the input and resulting image data back and forth between the GUI and GPU.

**Fig. 4A)** shows the front-end GUI display developed for this software, and **4** **B)** shows the block diagram of the reconstruction steps performed for an operator-selected temporal window, including the timing calculations for each reconstruction operation from the operator instructions to visualization. The total time required for the combined transfer of input and output data between MATLAB and GPU was constant at 0.18 + 0.01 seconds. Table 2 shows the average computation times at different reconstruction window sizes. The GPU implementation required a fixed 0.2 seconds for MATLAB-GPU interaction, 0.2–0.35 seconds for gridding—for a total of 0.4–0.55 seconds for visualization of the desired 2D slice from the operator-specified temporal window and slice. For CPU gridding with NUFFT, an initial preparation step of the appropriate k-space look-up table took 1.5–10.5 seconds before performing the gridding and visualization steps in a single step (1.2–1.8 seconds), which required ~3 times than the GPU implementation.

**Table 2:** Average Processing Times GPU vs CPU Methods.

Test Methodology	Total Computation Time (seconds)				
(#Samples × #Spokes × #Coils) #k-space points to grid	GPU-TRANSFER	GPU-GRID	GPU-TOTAL	CPU-PREP	CPU-GRID TOTAL
1.3 mm resolution Recon					
RecWin Size = 7; (464 × 98 × 8) = 363776 pts	0.18 ± 0.01	0.19 ± 0.02	0.37 ± 0.04	1.5 ± 0.1	1.2 ± 0.1
RecWin Size = 16; (464 × 224 × 8) = 831488 pts	0.18 ± 0.01	0.23 ± 0.02	0.41 ± 0.04	3.5 ± 0.4	1.4 ± 0.1
RecWin Size = 48; (464 × 672 × 8) = 2494464 pts	0.18 ± 0.01	0.35 ± 0.03	0.53 ± 0.04	10.5 ± 1.1	1.8 ± 0.2
Submillimeter resolution Recon					
RecWin Size = 48; (600 × 864 × 8) = 4147200 pts	0.19 ± 0.01	0.78 ± 0.08	0.96 ± 0.10	17.9 ± 1.8	2.8 ± 0.29

Significant speed-up in total processing time is observed compared to the NUFFT-based preparation implemented in MATLAB, which required as much as 13 seconds (10.5 for prep + 1.8 for gridding), and is therefore unsuitable for a flexible and retrospective reconstruction workflow for interactive visualization.

### In Vivo Imaging

All scans were completed successfully. The average navigator efficiency for the whole-heart coronary imaging across the eight subjects was 41 ± 11%. Table 3 reports the normalized vessel sharpness scores from the operator assessments, as well as its difference between the score from the standard window, while Table 4 reports the adjusted temporal window selected by each operator. Table 5 summarizes the SNR and CNR measurements.

**Table 3:** Vessel Wall Sharpness Scores.

Subj.	RCA (Root)	RCA (Mid)	RCA (Distal)	LAD
	[Standard Window Score; Manually Derived Window Score] (%) Repeated measurement in next row.			
1	0.37; 0.43	ND; 0.36*	ND; 0.22*	0.29; 0.30
	0.37; 0.43	ND; 0.37*	ND; 0.24*	0.30; 0.32
2	0.37; SAME	0.37; 0.42	0.24; 0.30	0.30; SAME
	0.33; SAME	0.38; 0.39	0.24; 0.30	0.27; SAME
3	0.34; 0.39	ND; 0.38*	ND; 0.31*	0.30; 0.40
	0.32; 0.37	ND; 0.39*	ND; 0.30*	0.32; 0.42
4	0.37; 0.39	0.31; 0.31	ND; ND	0.42; 0.39
	0.31; 0.38	0.28; 0.29	ND; ND	0.40; 0.38
5	0.36; 0.40	ND; 0.44*	0.21; 0.29	0.27; 0.37
	0.35; 0.38	ND; 0.42*	0.22; 0.28	0.27; 0.38
6	0.42; SAME	0.45; 0.49	0.29; SAME	0.41; SAME
	0.43; SAME	0.44; 0.46	0.30; SAME	0.41; SAME
7	0.28; SAME	—	—	ND; ND
	0.28; SAME	—	—	ND; ND
8	0.52; SAME	0.41; 0.47	ND; ND	0.42; SAME
	0.53; SAME	0.42; 0.49	ND; ND	0.44; SAME

(*)—Vessel was non-descript with the standard, but visualized with the manually window.

(SAME)—The standard window yielded good image quality after visual assessment.

(ND)—Vessel was non-descript.

(—)—Subject 7 exhibited having a small rudimentary right with left-dominance, thus the RCA-mid to distal end was not present. BOLD—reconstructions are shown in Fig. 5.

**Table 4:** Manually Selected vs Standard Temporal Window.

Subj.	Standard Window	RCA (Root)	RCA (Mid)	RCA (Distal)	LAD
	[Start of Window, End of Window.] (milliseconds) with respect to ECG trigger delay.				
1	[620, 740]	[583, 670]	[579, 630]	[592, 651]	[611, 717]
2	[620, 740]	SAME	[575, 639]	[583, 657]	SAME
3	[510,630]	[497, 571]	[492, 556]	[469, 543]	[573, 660]
4	[600,720]	[559, 679]	[559, 679]	ND	[609, 715]
5	[550,670]	[528, 625]	[509, 564]	[545, 632]	[505, 597]
6	[580,700]	SAME	[566, 658]	SAME	SAME
7	[670, 890]	SAME	—	—	ND
8	[650, 720]	SAME	[650, 714]	ND	SAME

(SAME)—The standard window yielded good image quality after visual assessment. (ND)—Vessel was non-descript.

(—)—Subject 7 exhibited having a small rudimentary right with left-dominance, thus the RCA-mid to distal end was not present.

**Table 5:** SNR and CNR Measurements.

Subj.	RCA (Root)	RCA (Mid)	RCA (Distal)	LAD
	[Myocardium SNR; Lumen SNR; Lumen-to-Myocardium CNR]			
1 (Standard)	8.6; 22.0; **13.4**	ND*	ND*	5.6; 13,9; **8.3**
(Adjusted)	6.7; 23.8; **17.1**	5.6; 21.8; **16.2**	7.3; 14; **6.7**	5.4; 17.6; **12.2**
2 (Standard)	7.2; 16.7; **9.6**	10.2; 21.9; **11.7**	8.4; 20.8; **12.4**	5.7; 15.2; **9.5**
(Adjusted)	SAME	4.4; 18.4; **13.9**	6.3; 16.5; **10.2**	SAME
3 (Standard)	4.5; 15.1; **10.6**	ND*	ND*	5.6; 17.9; **12.3**
(Adjusted)	5.1; 20.8; **15.7**	7.1; 17.1; **10.0**	7.2; 16.4; **9.2**	6.0; 20.6; **14.6**
4 (Standard)	10.1; 23.8; **13.7**	9.0; 27.5; **18.5**	ND	6.2; 22.1; **15.9**
(Adjusted)	7.6; 28.6; **21.0**	12.9; 30.0; **17.1**	ND	6.5; 24.7; **18.3**
5 (Standard)	13.0; 26.4; **13.3**	ND*	7.0; 14.9; **7.9**	4.1; 11.8; **7.7**
(Adjusted)	6.6; 22.2; **15.6**	4.5; 15.7; **11.3**	6.7; 16.8; **10.1**	6.2; 17.6; **11.4**
6 (Standard)	6.8; 36.3; **29.5**	12.8; 38.3; **25.5**	7.7; 19.7; **12.1**	5.5; 17.8; **12.3**
(Adjusted)	SAME	15.9; 56.2; **40.3**	SAME	SAME
7 (Standard)	9.4; 32.8; **23.5**	—	—	ND
(Adjusted)	SAME			ND
8 (Standard)	10.5; 41.1; **30.6**	13.5; 34.3; **20.8**	ND	8.8; 34.8; **26.0**
(Adjusted)	SAME	10.2; 46.0; **35.8**	ND	SAME

(*)—Vessel was non-descript with the standard, but visualized with the manually window.

(SAME)—The standard window yielded good image quality after visual assessment.

(ND)—Vessel was non-descript.

(—)—Subject 7 exhibited having a small rudimentary right with left-dominance, thus the RCA-mid to distal end was not present.

BOLD—Lumen-to-Myocardium CNR.

**Fig. 5** demonstrates examples of motion corrected vessel at the root of the RCA, and a distal RCA vessel recovered by the operator using the proposed approach. **Fig. 6** shows operator’s workflow to optimize the visualization at the mid-RCA region by manipulating the reconstruction’s temporal window in an interactive manner. In this case, the operator was able to fully recover the RCA that was non-descript with the initial window (left) by gradually adjusting the reconstruction window to that yielding optimal visualization with minimal blurring motion.

**Fig 5:**
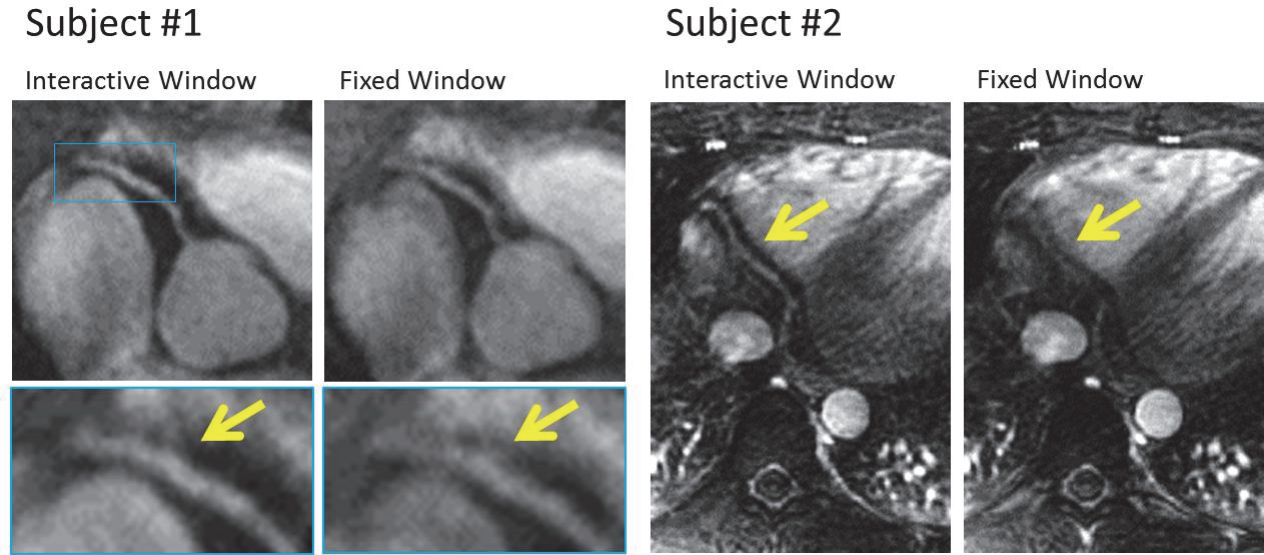
Examples of the impact of interactive vs. fixed selection of the trigger delay and window for visualization of RCA in two different subjects. In Subject #1, using the interactively selected window mitigates the temporal blurring seen in RCA images reconstructed using fixed trigger delay and window. In Subject #2, the distal region of RCA can be fully recovered by interactively adjusting the trigger delay and window size.

**Fig 6:**
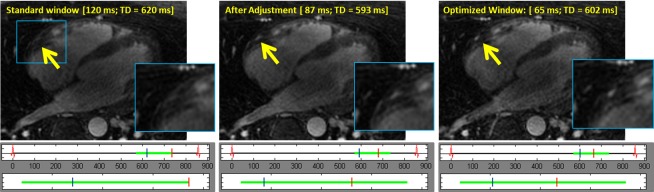
The operator’s workflow for optimal visualization of the mid-RCA region. In A) the images reconstructed from the fixed pre-determined window of 120 ms and trigger delay of 620 ms. The RCA cannot be visualized presumably due to cardiac motion. In B), the reconstruction window was adjusted to 87 ms with a TD of 593 ms and in C) a 65 ms reconstruction window with a TD of 602 ms, which results is in a motion-free clear visualization of the RCA. The operator scrolled through multiple slices (not shown) to verify the optimal reconstruction for the RCA.

Out of 30 examined artery regions, 9 were sufficiently well-visualized using the standard window, and operator interaction was performed in 19 regions. Of these, 14 regions (4 each in RCA-root, mid, and LAD, and 2 in RCA-distal) yielded a visible vessel with the standard window that was manipulated by the operator for better visualization, while 5 were fully visualized using the proposed method when it was non-descript for the standard window (eg. Fig 5–2, right). The lumen-to-myocardium CNR increased by 30±25% (n = 14; p < 0.005) after manual adjustment compared to the vessels depicted in the standard 120 ms window reconstructions. Three regions were not examined due to any visible artery region throughout the entire acquisition window. For the RCA root and LAD, the average sharpness scores were (manually selected vs standard 120 ms windows): RCA (Root) 0.40 ± 0.06 vs 0.38±0.07; (LAD) 0.36±0.06 vs 0.34±0.07. Repeated measurements yielded: RCA (Root) 0.39 ± 0.07 vs 0.37±0.08; (LAD) 0.37±0.06 vs 0.34±0.07. The vessel sharpness assessments were statistically significant and improved (P < 0.0005 for both measurements) in the 14 regions where the vessel region was visualized in the standard window, but necessitated interactive adjustment by the operator. The average sharpness score improvements in these regions were both 0.05 ± 0.04 for measurements 1 and 2.

## Discussion

In this study, we have demonstrated the feasibility of acquiring an enlarged acquisition window using a 3D radial stack-of-stars sampling scheme with a GA-derived customized angle for optimal k-space distribution to enable reconstruction of any flexible temporal subset of the acquisition window in a retrospective manner, allowing tailored reconstruction for different coronary artery branches and regions within the acquired 3D volume. An optimized rotation angle for the 3D k-space segmented sequence was derived from the GA radial sampling trajectory, allowing a flexible reconstruction with a minimal subset of at least 7 k-space spokes per heart-beat and a temporal resolution of 32 ms. A simulation was performed to report the optimal rotation angle for a fixed nTFE and nHB pair. A look-up-table was derived for the optimal GA/n angle as reported in Table 1, which provides a reasonable coverage of the k_x_-k_y_ planes with good image quality even under undersampled conditions accounting for both the optimal angle gap spacing and clustering of radial spokes within a sector of the k_x_-k_y_ plane. For rapid and interactive visualization of the data, a GPU-based reconstruction engine was combined with the GUI developed in MATLAB to enable an interactive assessment. When compared with the MATLAB-based NUFFT reconstruction method that is employed for offline non-Cartesian methods, the GPU-based radial reconstruction requires no dedicated preparation, and is therefore more suitable for on-the-fly visualization within 1 second even under computationally heavy conditions, which matches previously reported ranges [38]. While the gridding reconstruction using the NUFFT package in MATLAB performs sufficiently well for most offline radial gridding reconstructions at our site that are acquired with lower spatial resolutions, a more robust GPU gridding implementation enabled the real-time visualization approach required for the clinical assessment tool in this study.

The strength of the proposed combined acquisition and reconstruction approach lies in its ability to enable visualization of any slice of the whole-heart 3D volume in real-time from the time-sorted k-space data. The additional consideration of the temporal dimension in the reconstruction step enables a retrospective examination of any flexible subset of the enlarged acquisition window. Further scan time reduction can be gained by undersampling the radial stack-of-stars sequence by incorporating iterative reconstruction approaches such as parallel imaging [39] or compressed sensing [40].

There are several limitations to this study. First, the contrast mechanism throughout the enlarged acquisition window is not optimized in this study with respect to: a) fat suppression, and b) contrast optimization throughout the enlarged acquisition window. For fat suppression, we employ a conventional fat saturation preparation prior to the radial SSFP acquisition of the enlarged acquisition window. Thus, this approach can suffer from fat signal recovery, and was subsequently used with contrast enhancement. Combination with fat water separation methods, such as Dixon techniques [41], or using water selective imaging approaches, such as ATR [42] or wideband SSFP [43], can potentially address this at the expense of prolonged scan time. Finally, in this study, we only examined the RCA and the LAD in healthy subjects. A more detailed examination of our technique in 1) a larger study population, 2) in patients with suspected or known coronary artery disease, and 3) assessing all coronary vessels are warranted.

In conclusion, this study demonstrates the feasibility of a whole-heart coronary imaging approach with an enlarged acquisition window by which the trigger delay and acquisition window can be adjusted interactively to tailor the reconstruction of different coronary branch regions. The sub-optimal distribution of k-space spokes in a segmented radial stack-of-stars acquisition with different acquisition and reconstruction windows is first addressed by the proposed optimization approach, and a GUI tool is developed to enable a flexible GPU-based offline radial k-space reconstruction for interactive assessment of any desired temporal window from the 4D coronary k-space data.

## Appendix

Let Θ_0_ denote $\Theta_{\theta ,\;\text{nTFE}}^{\text{nHB},\;\text{nRCW}}$ for a fixed (*θ*, nTFE, nHB, nRCW) in a reconstruction window between [0, nRCW]. Then, any subsequent reconstruction window with an offset of *k* TRs can be denoted as [*k*, *k* + nRCW], denoted byΘ_*k*_. As the rotation angle is a fixed constant *θ* during the acquisition, Θ_*k*_=(Θ_0_ + *k*θ) *modulo* *π*, an element-wise addition, which is equivalent to a global rotation of the radial spokes by *kθ*. Subsequently, it can be seen that the angle gap $\Delta \Theta_{k}\equiv \Delta \Theta_{0}$, as this gap does not change over a global rotation. Hence, $\Delta \Theta_{\theta \;,\text{ nTFE, nHB, nRCW}}$ is invariant to the position of the reconstruction window, and can be simplified as a function that depends solely on nRCW regardless of its relative position within the acquisition window.
